# When “small” terms matter: Coupled interference features in the transport properties of cross-conjugated molecules

**DOI:** 10.3762/bjnano.2.95

**Published:** 2011-12-29

**Authors:** Gemma C Solomon, Justin P Bergfield, Charles A Stafford, Mark A Ratner

**Affiliations:** 1 Nano-Science Center and Department of Chemistry, University of Copenhagen, Universitetsparken 5, 2100 Copenhagen Ø, Denmark; 2 Department of Physics, University of Arizona, 1118 East Fourth Street, Tucson, AZ 85721, USA; 3 Department of Chemistry, Northwestern University, 2145 Sheridan Rd, Evanston, IL 60208, USA

**Keywords:** gDFTB, Hückel model, many-body effects, molecular electronics, quantum interference, thermoelectrics, topology

## Abstract

Quantum interference effects offer opportunities to tune the electronic and thermoelectric response of a quantum-scale device over orders of magnitude. Here we focus on single-molecule devices, in which interference features may be strongly affected by both chemical and electronic modifications to the system. Although not always desirable, such a susceptibility offers insight into the importance of “small” terms, such as through-space coupling and many-body charge–charge correlations. Here we investigate the effect of these small terms using different Hamiltonian models with Hückel, gDFTB and many-body theory to calculate the transport through several single-molecule junctions, finding that terms that are generally thought to only slightly perturb the transport instead produce significant qualitative changes in the transport properties. In particular, we show that coupling of multiple interference features in cross-conjugated molecules by through-space coupling will lead to splitting of the features, as can correlation effects. The degeneracy of multiple interference features in cross-conjugated molecules appears to be significantly more sensitive to perturbations than those observed in equivalent cyclic systems and this needs to be considered if such supernodes are required for molecular thermoelectric devices.

## Introduction

Destructive interference effects, such as nodes in the transmission function, are a signature of coherence and offer a possible avenue for tuning the transport properties of single-molecule junctions. While not present in all systems, destructive interference features are observed in many common systems. For example, in the *meta*-substituted Au–benzenedithiol–Au junction the π-electron transmission exhibits a node in the middle of the gap between the highest occupied molecular orbital and lowest unoccupied molecular orbital [1–13], although the total conductance is nonzero, as underlying σ-system transport dominates in the vicinity of the node [8–9]. Through careful design, interference effects can be perturbed by chemical modification or an external electric field [6,14], presenting myriad strategies to control the flow of charge through a molecular circuit. Thermoelectric effects are also strongly influenced by the presence of interference features, and enhancement is predicted in the vicinity of nodes and peaks [15–16]. In certain molecules composed of node-possessing subunits, multiple degenerate interference features may combine to form higher-order nodes (supernodes) and peaks whose thermoelectric enhancement scales as the order of the feature [16]. Supernode-possessing molecules also suppress current over a wide range of energy, suggesting that they may be an important step towards realizing useful molecular devices.

Previous work on supernodes focused on cyclic systems [16], but the transport properties of both cyclic [17–18] and acyclic [19–20] cross-conjugated molecules have also been predicted to exhibit interference features in experimentally relevant energy ranges. Here we investigate the transport through several acyclic cross-conjugated molecules and show that maintaining degenerate interference features in these systems may be challenging. As a basis for comparison, we use Hückel theory transport calculations to understand what can be expected from the topology alone. Using gDFTB and many-body calculations, we find that the order of the interference feature in acyclic systems can be strongly dependent upon through-space terms and electron correlations.

## Methods

Transport in a single-molecule junction is often described by using Green’s function approaches, where the elastic transmission is generally calculated as [21]

[1]
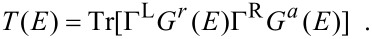


*G^r^*(*E*) is the retarded Green’s function of the junction at energy *E*, *G^a^*(*E*) is its conjugate transpose, and Γ^L^ and Γ^R^ are the broadening matrices describing the coupling to the left and right electrodes, respectively. In each of the three theoretical methods discussed below, the differences in the methods manifest themselves as differences in the Green’s functions. The gDFTB and molecular Dyson equation (MDE) many-body methods used in this article are explained in detail in [22] and [23], respectively. Here we simply provide a brief overview of the aspects most relevant to transport in the cross-conjugated systems investigated here.

### Hückel model calculations

**Figure 1:**
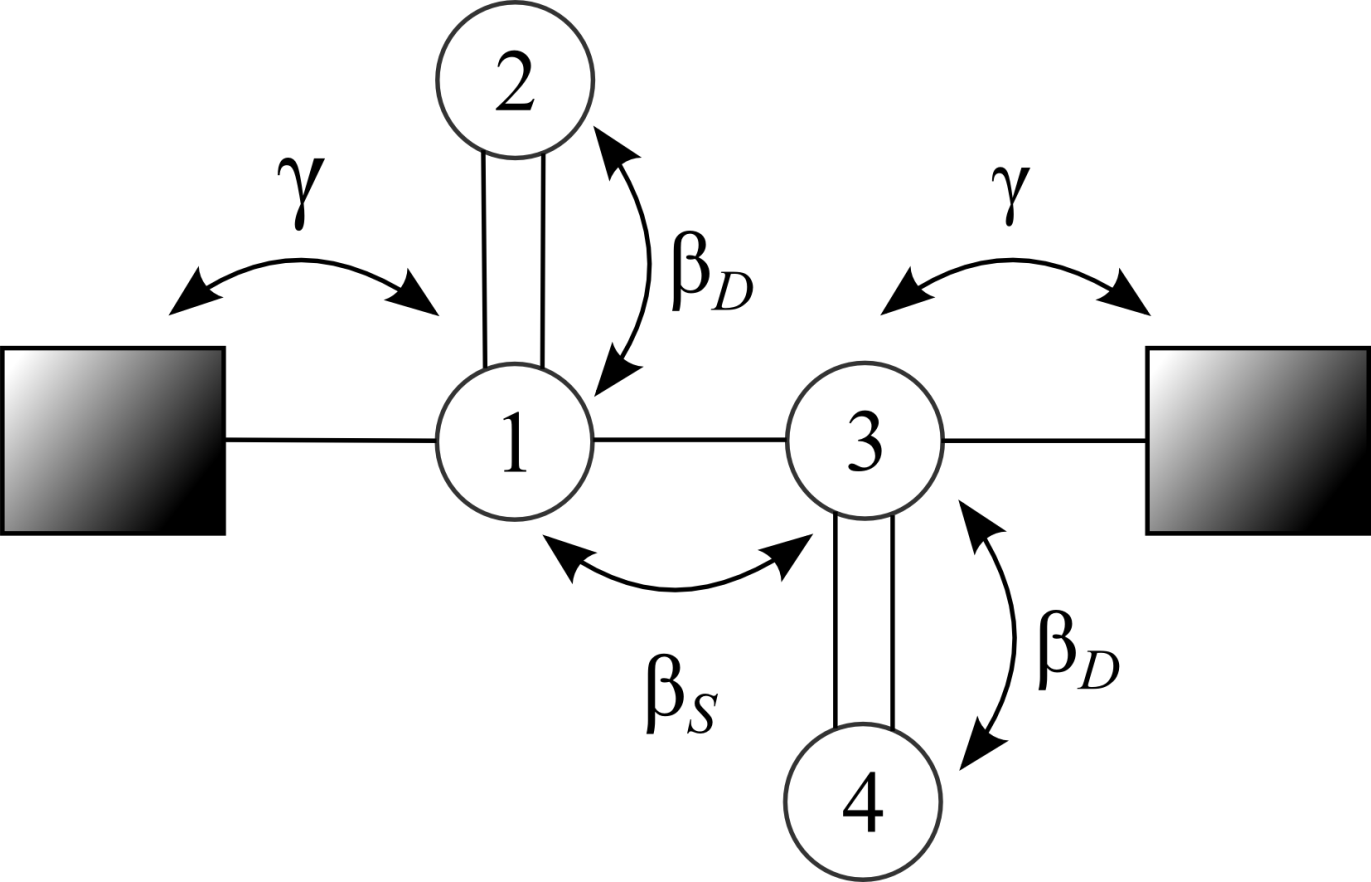
An example of a Hückel model for a four-site molecule, the numbering of the sites corresponds to the index in the Hamiltonian.

A simple multisite model Hamiltonian can be constructed by representing each relevant atomic orbital of the molecule by an energy *α*. Between chemically bonded nearest-neighbor sites there are coupling elements, β*_S_* and β*_D_*, depending on whether sites have single or double bonds between them. For example, in Figure 1, a four-site system is shown.

The Hamiltonian for this system is given by:

[2]
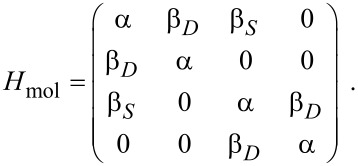


In all calculations shown here we set α = 0 eV, β*_S_* = −3 eV, and β*_D_* = −4 eV. We assume that only a single site couples to each electrode, with coupling strength γ = β*_S_*/3 = −1 eV. For this system we have:

[3]
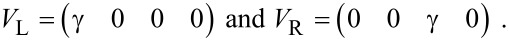


The transmission is then calculated by using the nonequilibrium Green’s function formalism assuming the wide-band limit for the density of states of the electrodes and setting

[4]
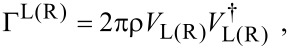


where *ρ* is the density of states of the electrode, which we set to 1/2π (*eV*)^−1^. This value is chosen to approximately reproduce the broadening seen in gDFTB calculations [9]. The purely imaginary tunneling self-energies are given by

[5]
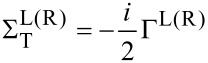


and the retarded Green’s function by

[6]
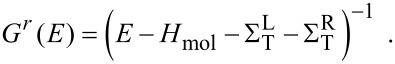


### gDFTB

The Hückel model calculations allow us to model the transmission through the carbon skeleton of a conjugated molecule, i.e., the component that we anticipate will dominate the transport properties in systems exhibiting destructive interference effects. Moving to atomistic simulations, it is necessary to add binding groups (here we use thiols) to bind the organic component to gold electrodes. The systems that we compare have the same underlying carbon skeleton but differ in the fact that the sulfur binding groups appear explicitly, rather than being effectively absorbed as part of the electrodes in the Hückel calculations.

The gDFTB method [22,24–25] allows us to calculate the transport with an atomistic model of the system, going beyond simple topology to include the through-space interactions that arise from the three-dimensional nature of chemical structures. Molecular geometries were obtained by optimizing the isolated molecule using Q-Chem 3.0 [26] with density functional theory employing the B3LYP functional and 6-311G** basis. The molecules were then chemisorbed (terminal hydrogens removed) to the FCC hollow binding site of a Au(111) surface with the Au–S bond length of 2.48 Å, taken from the literature [27].

Transport calculations by means of gDFTB construct the Green’s functions in the same way as the Hückel model calculations, that is simply by using the gDFTB Hamiltonian in place of the Hückel Hamiltonian. A wide-band approximation is also employed in which the density of states used to construct the self-energies is set to a value for bulk gold (1.9 eV ^−1^). No gold atoms were included in the extended molecule so that the symmetry of the molecule could be used to separate the transmission into σ- and π-components [28]. The electrode comprised a 6 × 8 atom unit cell with three layers in the transport direction, and periodic boundary conditions were used.

### Many-body calculations

The many-body problem of transport through a molecular junction is generally intractable and must be solved approximately. Often this is done perturbatively by using, for example, diagrammatic methods. Phrasing the perturbative series in terms of Green’s functions is advantageous since Dyson’s equation allows a finite number of physical processes to be calculated to infinite order. In contrast, perturbative methods based on the density matrix often sum all processes to finite order [29–33]. In this article, we consider off-resonant transport through small molecules in which the electrode–molecule coupling is on the order of the molecule’s charging energy. In this regime, the transport exhibits many nonperturbative effects (e.g., simultaneous charge quantization and quantum interference [34]) that cannot be properly described unless the processes are considered to infinite order.

For the many-body transport calculations presented in this article, we utilize a theory [23] based on the molecular Dyson equation (MDE) and nonequilibrium Green’s functions (NEGFs). In the MDE method, the Green’s function 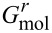
(*E*) of the junction is calculated by exactly diagonalizing the selected model Hamiltonian in the sequential-tunneling limit, including all excitations and charge states of the molecule. The effects of finite tunneling width are then included by using the equation-of-motion technique combined with diagrammatic perturbation theory for the Coulomb interactions. By using the molecular Dyson equation, the full Green’s function of the system may be written as [23]

[7]
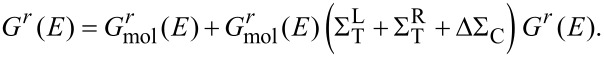


In general, the correction to the Coulomb self-energy ΔΣ_C_ must be found through NEGF methods [23]; however, in the elastic cotunneling regime ΔΣ_C_ = 0. The elastic transmission probability through a junction may be found by using Equation 1.

The molecular Green’s function 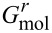 is found by exactly diagonalizing the molecular Hamiltonian. Represented in a basis of atomic orbitals, the Green’s function matrix elements are given by [23]

[8]
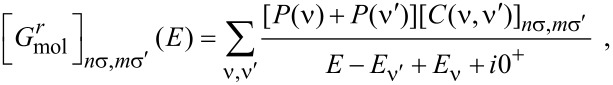


where *P*(*ν*) is the probability that the state *ν* of the nearly isolated molecule is occupied and *H*_mol_ 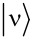 = *E_ν_* 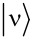 with the molecular Hamiltonian *H*_mol_. [*C*(*ν*,*ν*')]*_n_*_σ,_*_m_*_σ'_ are the many-body matrix elements given by

[9]
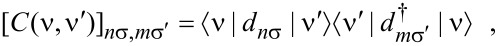


where *ν* and *ν*' label molecular eigenstates with different charge. Here *d_n_*_σ_ annihilates an electron of spin σ on the *n*th atomic basis function of the molecule. In linear response, *P*(*ν*) is given by the grand canonical ensemble.

The transport theory outlined above is generally applicable. Here we focus on single-molecule junctions and utilize a semiempirical Pariser–Parr–Pople (PPP) [35–37] π-electron Hamiltonian, which describes Coulomb interactions, π-conjugation and screening due to the σ-electrons and solvent in order to model the electronic degrees of freedom that are most relevant for transport [6,23,38]. The molecular Hamiltonian may then be written as follows

[10]
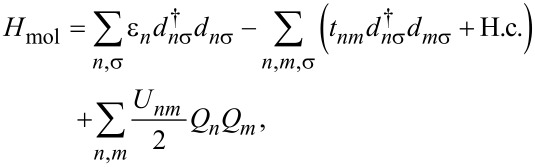


where ε*_n_* is the π-orbital energy, *t_nm_* is the hopping-matrix element between orbitals *n* and *m*, and *U_nm_* is the effective Coulomb interaction energy between orbitals *n* and *m* (where *n* and *m* may be equal) [39]. In correspondence with the Hückel and gDFTB calculations, we take the nearest-neighbor hopping-matrix elements *t_nm_* for single and double bonds to be 3 eV and 4 eV, respectively. The effective charge operator for orbital *n* is [6,15,40]

[11]
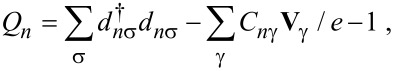


where *C_nγ_* is the capacitive coupling between orbital *n* and lead *γ*, *e* is the electron charge, and **V***_γ_* is the voltage on electrode *γ*. The “−1” in the charge operator sets the midgap energy to zero. For simplicity, the atomic basis orbitals are taken to be orthonormal in our calculations, so that the anticommutator 
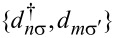
 = *δ_nm_δ*_σσ'_. The effective Coulomb interaction energies for π-conjugated systems can be written as an Ohno [41] potential with dielectric ε [38]

[12]
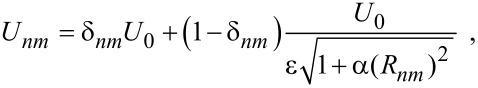


where *U*_0_ is the on-site Coulomb repulsion, *α* = (*U*_0_/14.397 eV)^2^, and *R_nm_* is the distance between orbital centers *n* and *m* in angstroms. Here we use *U*_0_ = 8.9 eV and ε = 1.28 [38]. The phenomenological dielectric constant ε accounts for screening due to both the σ-electrons and any environmental considerations, such as nonevaporated solvent [38]. Calculations presented here were performed by using a chosen basis with frozen atomic nuclei whose positions were taken from DFT calculations of the same junctions.

## Results

In conjugated molecules, including cross-conjugated molecules, the π-electron system dominates many of the observed physical properties. Consequently, simple descriptions of this itinerant electronic system, such as those provided by Hückel theory, can often capture a large part of the transport properties [9]. In cases where either the σ-system dominates, or where electron–electron interactions that are not included in the Hückel model calculation become important, these models will obviously break down.

For cyclic molecules, Luttinger’s theorem ensures [6,16] the efficacy of (effective) single-particle theories, such as DFT and Hückel theory, to predict interference features at the Fermi energy. However, there is no analogous theorem for cross-conjugated molecules, making them an interesting subject for investigation. Here we use Hückel, gDFTB and a many-body MDE theory to calculate the transport through each molecular junction and to determine the effects of topology, through-space coupling and interactions beyond the mean-field.

### The molecules considered

**Figure 2:**
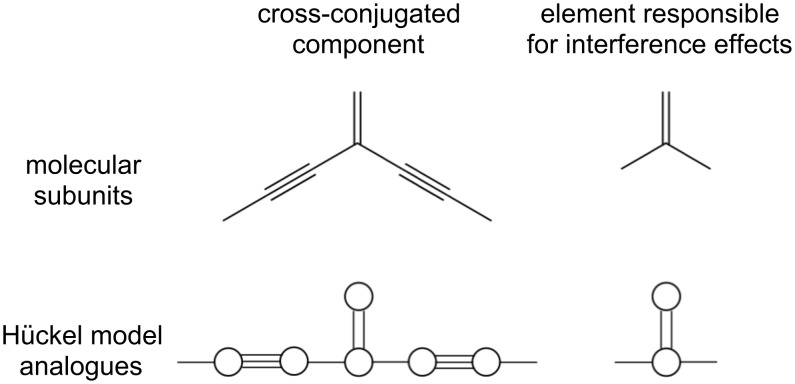
Examples of molecular structures (top) and the corresponding model systems (bottom) for true cross-conjugated structures (left) and the minimal systems (right) necessary to reproduce the characteristic interference features of the cross-conjugated structures in the electronic transmission.

In this paper we study a series of molecular systems based on acyclic cross-conjugated molecules. According to Phelan and Orchin, “A cross-conjugated compound may be defined as a compound possessing three unsaturated groups, two of which although conjugated to a third unsaturated center are not conjugated to each other” [42]. An example of a cross-conjugated subunit is shown on the left of Figure 2 as both a chemical structure and the model system that we can use in a Hückel model calculation. Cross-conjugated molecules have been shown to exhibit destructive interference features in the electronic transmission near the Fermi energy [19]; however, only the single unsaturated site in the center is necessary to produce these features. Consequently, the minimal systems required in order to study interference effects in these types of acyclic compounds are those shown on the right in Figure 2. While these systems are not strictly cross-conjugated according to the Phelan-Orchin definition, we will refer to them as cross-conjugated as they contain the essential elements needed to produce the transport signatures of cross-conjugation.

The number of interference features that can be expected in the transmission will scale with the number of the minimal units, exhibiting interference effects, that appear in the structure. We will also consider molecules with multiple interference features arising from multiple subunits of the type shown on the right of Figure 2. Again while these systems are not strictly cross-conjugated by definition, they nevertheless again contain the interference features characteristic of cross-conjugated systems and will be referred to as such.

### Hückel model systems: Topology and interactions through bonds

**Figure 3:**
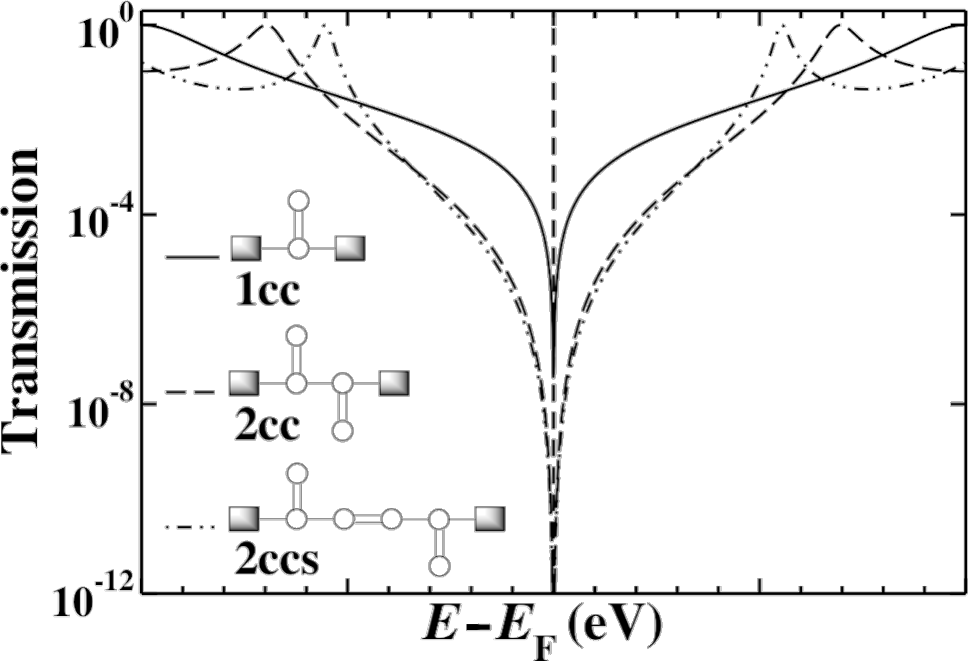
The transmission through three model cross-conjugated molecules calculated using Hückel theory. In each case, a single destructive interference feature (node) is evident at the Fermi energy. The order of the node increases with the number of cross-conjugated units from quadratic to quartic.

Consider three simple cross-conjugated molecules, shown as the inset to Figure 3. The smallest system (“1cc”) models a single cross-conjugated unit bound between two electrodes, the middle system two cross-conjugated units (“2cc”) and the largest system two cross-conjugated units separated by an extended conjugated bridge (“2ccs”). The Hückel Hamiltonian only contains information about the topology, so while we draw the larger systems with one cross-conjugated unit up and one down there would be no difference in the Hamiltonian if we instead wanted to model the system where both units were pointing in the same direction.

The transmission spectra of the 1cc, 2cc and 2ccs junctions are shown in Figure 3, calculated by means of Hückel theory. All three junctions exhibit a transmission node when *E* = *E*_F_ (here set to zero), although the nature of the nodes appears to be different in each case. Using Equation 1 with the Hückel Green’s function, Equation 6, we find that the transmission function of the 1cc junction approaches zero near the Fermi level, quadratically with respect to energy. Performing the same calculation for the 2cc molecule, composed of two cross-conjugated units, we find that the node is quartic. In both cases, this behavior has been detailed previously in similar systems in which the explicit form of the transmission around the node was detailed [15]. We also find a quartic node for the 2ccs junction. These higher order nodes are signatures of multiple degenerate interference features (supernodes). Such supernodes are potentially of technological importance, since the thermoelectric response may be significantly enhanced by their presence [16].

In molecules composed of cyclic components, such as polyphenylethers, single-particle notions, such as a Fermi surface, are protected by Luttinger’s theorem [43]. Therefore, very high-order nodes are predicted to exist for molecules composed of many rings [16]. At the Hückel level, we find an analogous situation in which the quadratic node of the 1cc subunit can be used to generate higher-order nodes, when connected appropriately. In the sections that follow, we investigate this result further by including interactions at both the mean-field and many-body level.

### gDFTB model systems: Interactions beyond π-bonds

Moving to an atomistic gDFTB model for these systems increases the number and changes the nature of the coupling terms that are included. In gDFTB calculations, both second and third nearest-neighbor Hamiltonian matrix elements are nonzero, and obviously these through-space terms will change if the molecular structure is varied in such a way that modifies these distances. The transmission through the smallest system (1cc) was published previously [19] and agrees well with the Hückel model calculations [44]. When we consider 2cc, however, the situation is not the same.

**Figure 4:**
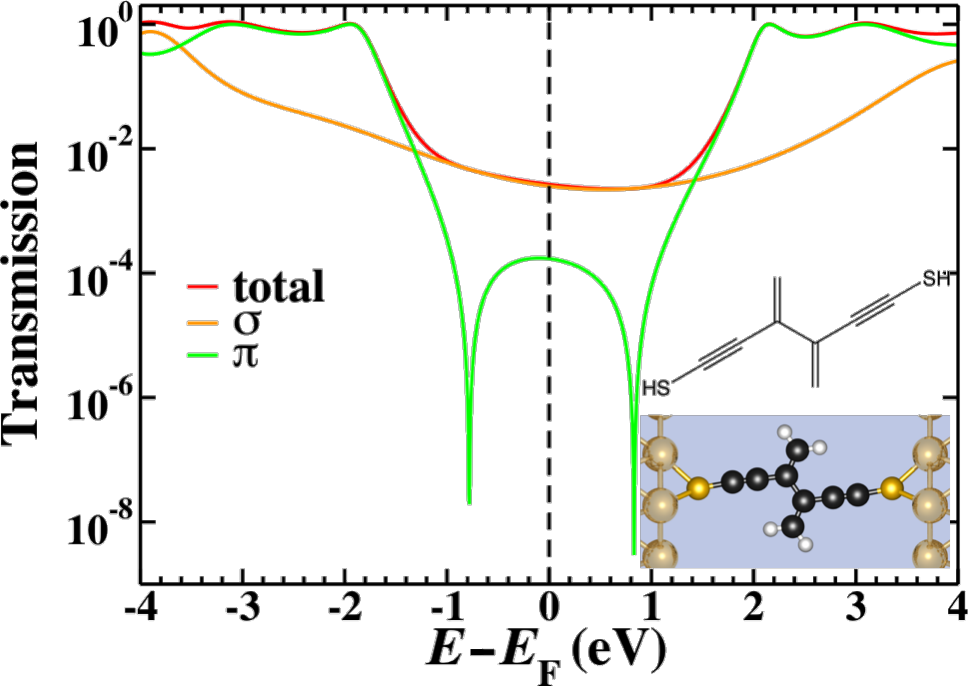
The chemical structure (inset top), space-filling model (inset bottom) and transmission for the 2cc system calculated by using gDFTB. The total transmission (red) is plotted along with the σ- (orange) and π- (green) components. The π- transmission exhibits two interference features separated by approximately 1.6 eV as opposed to the single interference feature observed in Figure 3.

Figure 4 shows the 2cc model we use in gDFTB. We extend the central 2cc unit with triple-bond spacer groups in order to ensure that the only interaction between the electrodes and the 2cc component occurs “through-bonds” rather than through any unintended “through-space” interactions “short-circuiting” the system. This modification extends the conjugation length and changes the position of the resonances; however, it has no bearing on the position of the interference features.

The total transmission as well as the σ and π components (separated using the symmetry plane) of a 2cc junction are shown in Figure 4. The interference features modeled in the Hückel calculations are only present in the π-component, but in this case the degeneracy of the interference features is lifted and both features are visible, symmetrically 0.8 eV above and below the Fermi energy. As in other small molecule systems, the sharp interference features are not visible in the total transmission as the σ transport is sufficiently high as to dominate across a large energy range. If chemical modification of the molecule extended the system to such an extent that the σ-transmission dropped below the π-transmission near the Fermi energy, the two split interference features would be revealed. Consequently, a system such as this would not behave as expected based on Hückel model calculations.

**Figure 5:**
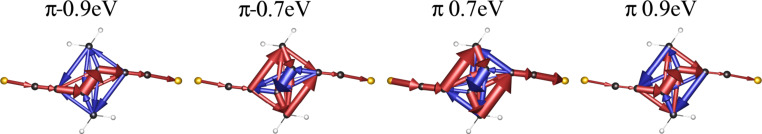
The local π-transmission (contributions to the transmission between pairs of atoms) for the species in Figure 4 either side of the two interference features, showing the complex ring-reversals of the coupled cross-conjugated units either side of the two interference features.

A method to inspect visually the contributions to the transmission in terms of coupling elements in the molecule, is to examine the local transmission through the system [45]. With this method, it is possible to separate the contributions to the transmission in terms of the contributions between atom pairs, providing insight into the through-bond and through-space interactions that dominate. Previous work [45] on the local transmission in the vicinity of the interference feature in 1cc highlighted the significance of through-space terms in this region, and to that extent it is perhaps not particularly surprising that interference features may be coupled in this way when multiple units are placed in such close proximity as they are in 2cc. The proximity of the two cross-conjugated units means that the second nearest-neighbor terms effectively couple one side chain to the base of the other cross-conjugated unit. Indeed this is reflected in the local transmission for this system as shown in Figure 5. We have seen previously [45] that interference features are characterized by ring-current reversals in the local transmission; by this we mean cyclic patterns in the local transmission with opposite handedness on either side of the interference feature moving along the energy axis. The ring-current reversals that characterize interference features in the gDFTB calculations of 2cc have a complex pattern across both side groups, directly illustrating how non-nearest-neighbor coupling terms can influence the nature of the interference features.

In addition to the second-nearest-neighbor terms, the third-nearest-neighbor terms also show significant contribution to this system. Similar terms were observed in the local transmission through an alkane with a gauche defect [45]. All of these non-nearest-neighbor terms are quite small compared with their nearest-neighbor counterparts (approximately 1 order of magnitude smaller), so it would be generally safe to assume that their role is not highly significant. In this case, however, the precise balance of the interactions that control the energy at which the interference features are observed is very sensitive to these small terms.

**Figure 6:**
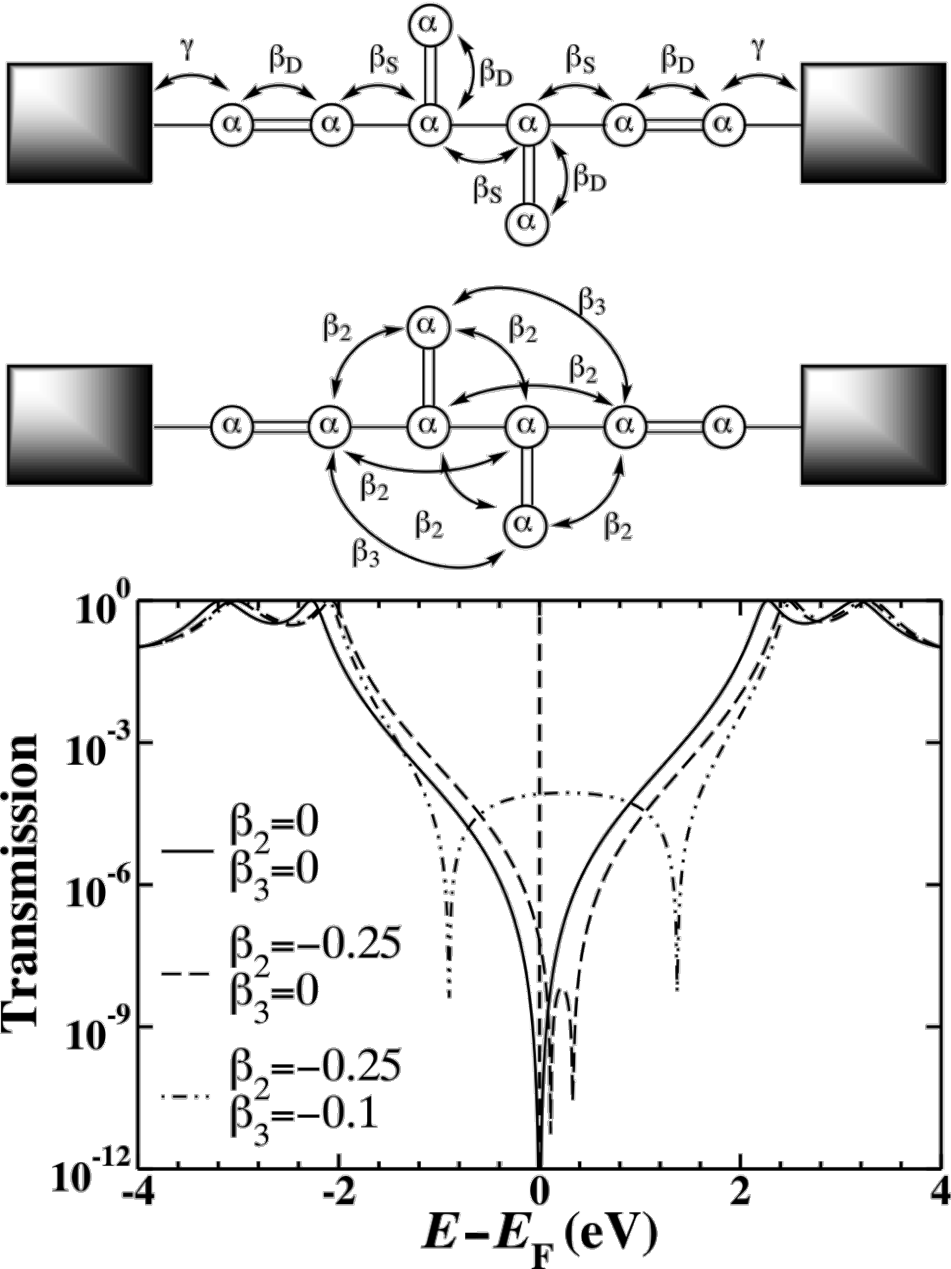
A modified Hückel model for 2cc with first- (solid), second- (dashed) and third- (dashed-dot) nearest-neighbor interactions included. The large, symmetric splitting of the interference features observed in the gDFTB calculations is only recovered when the very small third-nearest-neighbor coupling elements are included.

The differing role of the second- and third-nearest-neighbor terms can be clarified by constructing a modified Hückel model that includes these terms. The coupling elements that are included are illustrated diagrammatically in Figure 6. We set β_2_ = −0.25 eV and β_3_ = −0.1 eV; these values were chosen to be of approximately the same magnitude as similar elements in the gDFTB Hamiltonian. The transmission in Figure 6 shows that while the addition of second-nearest-neighbor interactions induces a partial, asymmetric splitting of the interference features, the large, symmetric splitting only arises with the inclusion of third-nearest-neighbor terms.

**Figure 7:**
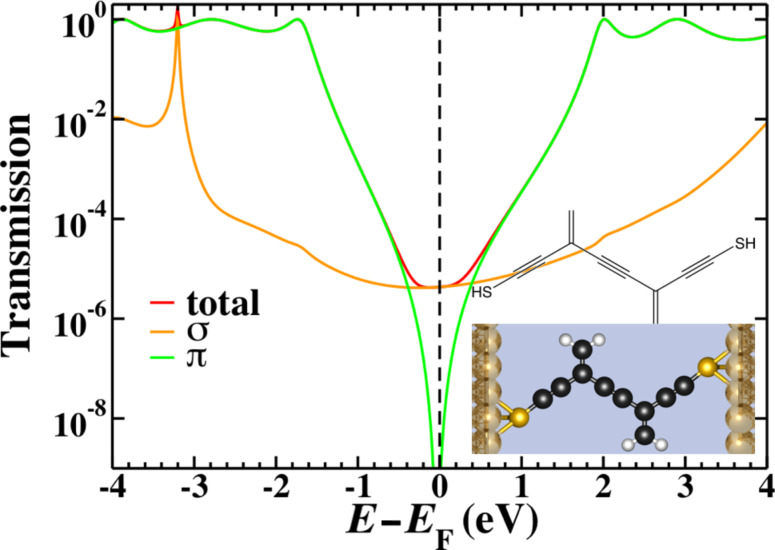
The chemical structure (inset top), space-filling model (inset bottom) and transmission for the 2ccs system calculated by using gDFTB. The total transmission (red) is plotted along with the σ- (orange) and π- (green) components. The π-transmission exhibits a single interference feature (which we interpret as two degenerate interference features) near the Fermi energy.

The role of second- and third-nearest-neighbor coupling terms in the splitting of the interference features means that extending the system to 2ccs can return the degeneracy of the interference features. Indeed that is what is observed in the transmission for 2ccs as shown in Figure 7 and was observed in similar systems previously [19] where it was noted that the site energies of the side groups controlled the position of the interference features.

**Figure 8:**
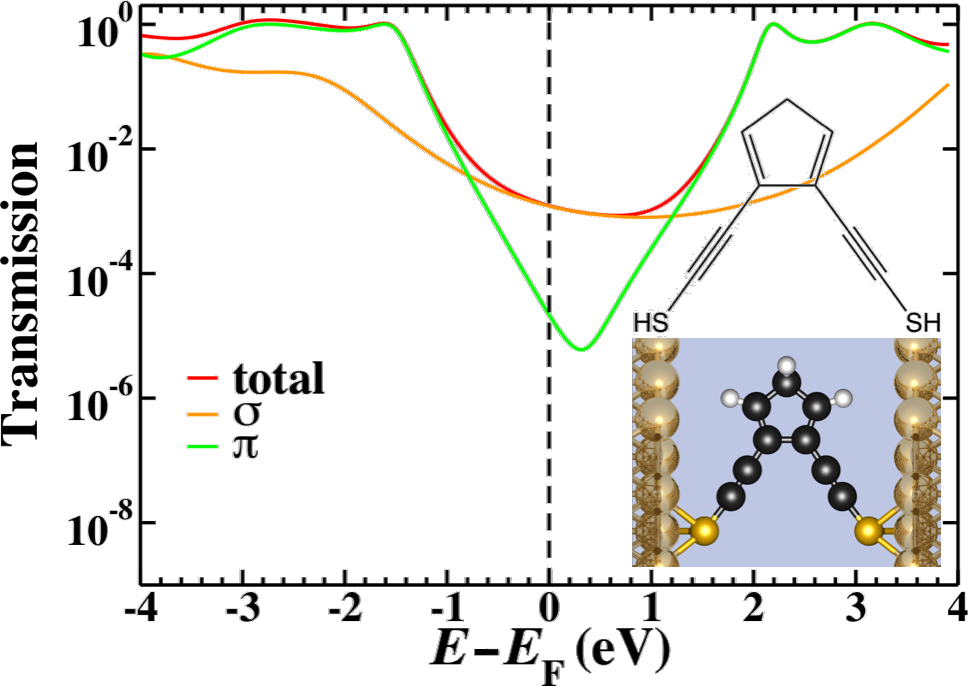
The chemical structure (inset top), space-filling model (inset bottom) and transmission for the 2cc-type system with hyperconjugative coupling between the cross-conjugated units as calculated by using gDFTB. The total transmission (red) is plotted along with the σ- (orange) and π- (green) components. The π-transmission exhibits degenerate interference features, while the true zero is lost through the hyperconjugative coupling.

As a further illustration of the effects of coupling between cross-conjugated units we can consider the molecule shown in Figure 8. This system introduces hyperconjugative coupling between the two cross-conjugated units but also removes the third-nearest-neighbor terms that induced the splitting in 2cc. The side chains of the cross-conjugated units are placed in closer proximity and the hyperconjugative interaction would seem to couple them more strongly; however, the transmission shown in Figure 8 shows no sign of split interference features. The π-transmission exhibits a single minimum, but the true zero that characterizes many interference effects is lost through the hyperconjugative coupling.

As an aside, the presence of the interference feature in this system cannot be established by ring-current reversals as the two successive reversals return the currents to their original orientation. The characteristic ring-current reversals associated with interference features can be seen in Figure 5, as in that case the separated interference features mean that the reversed regime can be seen between −0.7 eV and 0.7 eV.

### Model systems with many-body effects: Charge–charge correlations

Although electronic many-body effects are not small, their effect on transport may be minimal in certain experimentally relevant regimes. As we have seen, even weak through-space tunneling interactions in molecules composed of multiple cross-conjugated units can lift the degeneracy of the supernode predicted by Hückel theory. In this section, we use the MDE many-body theory [23] to investigate the effect of charge–charge correlations on the transport.

**Figure 9:**
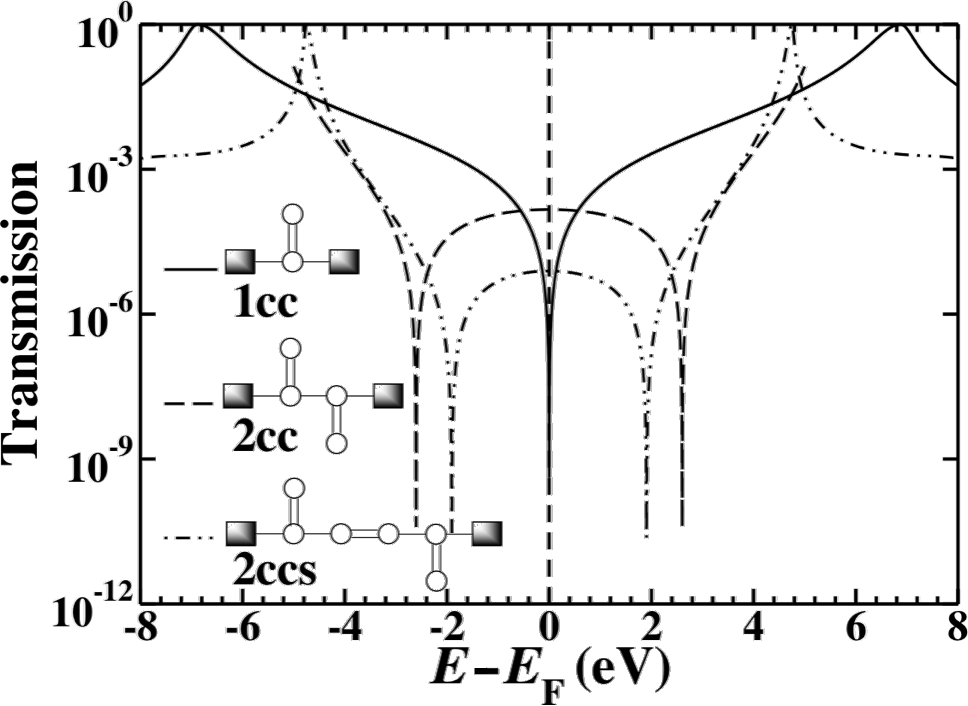
The same systems as calculated in Figure 3, but calculated by using the MDE many-body method. In both 2cc and 2ccs the interference features are split due to correlation effects since Coulomb interactions are always “through-space”. In these calculations only the nearest-neighbor tunneling elements are included in the electronic coupling. Notice that the node splitting is symmetric about *E* = *E*_F_ .

The transmission spectra through the π-orbitals of 1cc, 2cc and 2ccs based junctions calculated by using the MDE many-body theory (using the Hamiltonian of Equation 10) are shown in Figure 9, and no supernodes are observed. Qualitatively, the splitting of the central supernode in the 2cc and 2ccs junctions is similar to what was seen with the inclusion of through-space tunneling terms in the Hückel and gDFTb theories. However, quantitatively the splitting is much larger in the MDE spectra with values of ~5.22 eV and ~3.76 eV for the 2cc and 2ccs junctions, respectively. Only nearest-neighbor coupling elements were included in the MDE calculations (β_2_ = β_3_ = 0 eV), so the splitting of the nodes in the 2cc and 2ccs in Figure 9 is purely a consequence of the electron–electron Coulomb interactions.

In the acyclic 2cc system, the MDE transmission spectrum possesses two quadratic nodes rather than a single quartic supernode. Based on the Hückel and gDFTB calculations, one may predict that without through-space coupling elements, the antiresonance (node) of each 1cc subunit of the 2cc molecule would combine to produce a supernode. However, this is not the case as the Coulomb interactions are always through-space. In this system there is an avoided crossing between the two 1cc antiresonances mediated by the long-range Coulomb interactions instead of the short-range tunneling interactions. These terms, given by Equation 12, are roughly an order of magnitude larger than the through-space tunneling, and consequently the node splitting observed in the many-body spectrum is much larger than what is seen in the Hückel or gDFTB results.

The existence of supernodes in molecules composed of multiple *meta*-substituted benzene units [16] appears to be protected by Luttinger’s theorem and the degeneracy of the interference features should be maintained, although exact many-body calculations for such molecules are currently prohibitively difficult. In contrast, no such theorem exists for cross-conjugated molecules and the degeneracy of transmission nodes may be lifted by interactions. There may be instances where the splitting of interference features is desirable, and in those contexts it seems that cross-conjugated molecules offer additional flexibility over multiply cyclic molecules. For thermoelectrics, however, where thermoelectric enhancement is related to the order of the supernode [16], these results suggest that devices constructed from multiple cyclic units may be preferable.

## Conclusion

The sensitivity of destructive interference features to perturbations can be seen as both a strength and a weakness of these effects for the control of electron transport in molecules. On the one hand it makes these effects extremely amenable to tuning, either chemically or electrically, and thus offers a convenient option for control. On the other hand, various theoretical approaches can differ significantly, both qualitatively and quantitatively, in where they predict interference features to lie. Simple predictive schemes [46–47] based on single-particle models have been developed to provide a quick answer as to whether a particular topology will result in interference features in the HOMO–LUMO gap, but “the devil is in the details”. In many molecules the HOMO–LUMO gap is on the order of a few electronvolts; however, even a small shift of ~0.5 eV away from the Fermi energy can mean that an interference feature has no bearing on the low-bias conductance. Here we show that “small” terms in the Hamiltonian can shift and change interference features considerably, and these types of effects need to be considered when designing interference-based molecular devices.

For small bias voltages, the room-temperature transport through many small, strongly-coupled, single-molecule junctions is predominantly elastic. In the vicinity of a node in the elastic transmission, other contributions to transport, e.g., from the σ-systems, inelastic processes, etc., may become physically relevant since the nodes of each contribution will not necessarily coincide. Recently, calculations of transport through biphenyl-based molecular junctions suggest that, for sufficiently large bias voltages, interference features in the elastic transport may be obscured by inelastic (phonon-assisted) contributions [48]. Although the exact magnitude of the inelastic component in acyclic cross-conjugated molecules is not known, the contribution to the transport will simply be additive, as it is in the case of the σ-system transport.

From a theoretical standpoint, these systems offer an excellent opportunity to discriminate between theoretical methods. When methods differ, the predicted conductance can change by orders of magnitude and this clearly provides a useful tool with which to probe the significance of the various approximations made in each case. Measuring the very low conductance predicted in cases where destructive interference effects dominate may not be trivial, but it is also clear that these features may result in dramatic thermoelectric properties and these may be much more amenable to comparison with theoretical predictions.
